# Mouse Embryonic Stem Cells Expressing GDNF Show Enhanced Dopaminergic Differentiation and Promote Behavioral Recovery After Grafting in Parkinsonian Rats

**DOI:** 10.3389/fcell.2021.661656

**Published:** 2021-06-22

**Authors:** Rolando Lara-Rodarte, Daniel Cortés, Karla Soriano, Francia Carmona, Luisa Rocha, Enrique Estudillo, Adolfo López-Ornelas, Iván Velasco

**Affiliations:** ^1^Instituto de Fisiología Celular – Neurociencias, Universidad Nacional Autónoma de México, Mexico City, Mexico; ^2^Laboratorio de Reprogramación Celular, Instituto Nacional de Neurología y Neurocirugía “Manuel Velasco Suárez,” Mexico City, Mexico; ^3^Departamento de Farmacobiología, Centro de Investigación y de Estudios Avanzados (Cinvestav), Mexico City, Mexico; ^4^División de Investigación, Hospital Juárez de México, Mexico City, Mexico

**Keywords:** glial cell line-derived neurotrophic factor, Parkinson’s disease, dopaminergic neurons, 6-hydroxydopamine, dopamine release

## Abstract

Parkinson’s disease (PD) is characterized by the progressive loss of midbrain dopaminergic neurons (DaNs) of the *substantia nigra pars compacta* and the decrease of dopamine in the brain. Grafting DaN differentiated from embryonic stem cells (ESCs) has been proposed as an alternative therapy for current pharmacological treatments. Intrastriatal grafting of such DaNs differentiated from mouse or human ESCs improves motor performance, restores DA release, and suppresses dopamine receptor super-sensitivity. However, a low percentage of grafted neurons survive in the brain. Glial cell line-derived neurotrophic factor (GDNF) is a strong survival factor for DaNs. GDNF has proved to be neurotrophic for DaNs *in vitro* and *in vivo*, and induces axonal sprouting and maturation. Here, we engineered mouse ESCs to constitutively produce human GDNF, to analyze DaN differentiation and the possible neuroprotection by transgenic GDNF after toxic challenges *in vitro*, or after grafting differentiated DaNs into the striatum of Parkinsonian rats. GDNF overexpression throughout *in vitro* differentiation of mouse ESCs increases the proportion of midbrain DaNs. These transgenic cells were less sensitive than control cells to 6-hydroxydopamine *in vitro*. After grafting control or GDNF transgenic DaNs in hemi-Parkinsonian rats, we observed significant recoveries in both pharmacological and non-pharmacological behavioral tests, as well as increased striatal DA release, indicating that DaNs are functional in the brain. The graft volume, the number of surviving neurons, the number of DaNs present in the striatum, and the proportion of DaNs in the grafts were significantly higher in rats transplanted with GDNF-expressing cells, when compared to control cells. Interestingly, no morphological alterations in the brain of rats were found after grafting of GDNF-expressing cells. This approach is novel, because previous works have use co-grafting of DaNs with other cell types that express GDNF, or viral transduction in the host tissue before or after grafting of DaNs. In conclusion, GDNF production by mouse ESCs contributes to enhanced midbrain differentiation and permits a higher number of surviving DaNs after a 6-hydroxydopamine challenge *in vitro*, as well as post-grafting in the lesioned striatum. These GDNF-expressing ESCs can be useful to improve neuronal survival after transplantation.

## Introduction

Parkinson’s disease (PD) is the second most common neurodegenerative disorder surpassed only by Alzheimer’s disease. It is characterized by the progressive loss of dopaminergic neurons (DaNs) in the *substantia nigra pars compacta* (SNpc) and the concomitant denervation of the dorsal striatum (Poewe et al., 2017). Loss of this highly specialized type of neurons results in severe depletion of dopamine (DA) levels in caudate-putamen and is responsible for the pathophysiological features in the disease such as tremor at rest, bradykinesia, and rigidity (Dauer and Przedborski, 2003). Current pharmacological treatments either involves the administration of dopamine receptors agonists or favors DA biosynthesis by supplementation of its precursor L-3,4-dihydroxyphenylalanine (L-DOPA), which is effective in some cases to improve motor symptoms but with associated side effects such as dyskinesias and on-off states. Unfortunately, such treatments lose effectiveness in the long term, since dopaminergic neurodegeneration continues (Lloyd et al., 1975; Hefti et al., 1981; Cenci, 2014). In fact, these treatments do not fully restore the normal release of DA in the brain and affect other systems that require this neurotransmitter, causing side effects (Cenci, 2014).

Survival of DaNs is a key aspect in the installment and progression of PD. A group of neurotrophic factors and neurotrophins such as Glial cell line-derived neurotrophic factor (GDNF), Neuturin (NRTN), and Brain-derived Neurotrophic Factor (BDNF) increase survival of DaNs (Nasrolahi et al., 2018). There are reports that neurotrophic molecules change in the brain of PD patients, specifically, a decrease in BDNF in the *substantia nigra* (Mogi et al., 1999) and a moderate increase of GDNF in the putamen (Mogi et al., 2001). Clinical trials tested the idea of promoting DA neuron survival in the brain by direct infusion of GDNF (Whone et al., 2019a, b) and NRTN (Olanow et al., 2015); however, the overall conclusion is that these neurotrophic molecules were not enough to promote recovery from the classical symptoms and presented important side effects. Experimentally, the use of proteins that increase neuronal viability suggests that neurotrophic factors can prevent neurodegeneration after 6-hydroxydopamine (6-OHDA) (Kearns and Gash, 1995; Åkerud et al., 2001).

Cell therapy with DA neurons from human fetal mesencephalic tissue has been tested in PD patients with variable outcomes (Lindvall et al., 1992; Kordower et al., 1995; Freed et al., 2001; Olanow et al., 2003). Since the availability of fetal tissue is limited, alternative sources to obtain DA neurons have been devised. Embryonic stem cells (ESCs) respond to developmental signaling to differentiate into DA neurons. Such differentiated progeny provides behavioral recovery after grafting in the striatum of experimentally lesioned animals (Chung et al., 2002; Kim et al., 2002; Rodríguez-Gómez et al., 2007; Kriks et al., 2011; Tripathy et al., 2013). However, transplanted DaNs have shown suboptimal survival and poor innervation of the host brain. Additionally, survival, growth, and function of transplanted DaNs are reduced in aged rats due to less trophic support from the host brain (Collier et al., 1999). To circumvent the lack of trophic support, researchers have grafted neural stem cells that express GDNF together with ventral mesencephalic neurons in the brain of lesioned rats and improve recovery and survival (Deng et al., 2013). Recently, a similar strategy expressing GDNF through viral transduction, either before or after transplantation, showed that delayed GDNF increased survival and innervation of grafted DA cells (Gantner et al., 2020).

Cultured cells offer the possibility of effective manipulation *in vitro* prior to transplantation. Here, we engineered mouse ESCs (mESCs) to constitutively produce human GDNF, to analyze DA differentiation and the possible neuroprotection by transgenic GDNF after toxic challenges *in vitro* or after grafting into the striatum of rats lesioned with 6-OHDA.

## Materials and Methods

### Cell Lines

R1 mESCs were transduced with lentivirus containing the empty vector, which are designated control (CTRL-ESC), or with hGDNF (GDNF-ESC), as previously reported (Cortés et al., 2016). Cells were genotyped by end-point PCR for hGDNF detection using a 30-cycle program at 59°C. Primers (in 5′–3′) for transgenic hGDNF: Forward (Fwd), AACAAATGGCAGTGCTTCCT and Reverse (Rev), AGCCGCTGCAGTACCTAAAA; for GAPDH, used as a positive control: Fwd, ATCACCATCTTCCAGGAGCG and Rev, CCTGCTTCACCACCTTCTTG.

### *In vitro* Differentiation of ESCs to DaNs

We used CTRL-ESC and GDNF-ESC, which have been proved to produce spinal motor neurons (Cortés et al., 2016). The dopaminergic differentiation protocol, which consists of five stages, was performed as previously reported (Lee et al., 2000). Briefly, CTRL-ESC and GDNF-ESC were cultured on gelatin-coated tissue cultured plates in the presence of Leukemia Inhibitory Factor in Knockout DMEM medium (Gibco, Carlsbad, CA, United States) supplemented with 15% ESC cell-tested fetal bovine serum (FBS). For the second stage, cells were dissociated with 0.05% Trypsin solution (Gibco) and plated onto bacterial dishes to induce formation of floating embryoid bodies (EBs) for 4 days. EBs were recovered and seeded onto adherent tissue culture plates in serum-free Insulin-Transferrin-Selenite medium (ITS) supplemented with 5 μg/ml Fibronectin (Invitrogen, Carlsbad, CA, United States) for 6 days, when migration of cells out of the EBs was evident. Expansion of Nestin-positive cells (stage 4) was initiated by seeding the single-cell suspension in glass coverslips precoated with 15 μg/ml poly-L-ornithine (Sigma, St Louis, MO, United States) and 1 μg/ml Fibronectin in N2 medium (Gibco) containing 10 ng/ml of basic fibroblast growth factor (bFGF), 100 ng/ml fibroblast growth factor 8 (FGF-8), and 100 ng/ml of human Sonic Hedgehog (R&D Systems, Minneapolis, MN, United States) during 4–6 days. Terminal differentiation at stage 5 was induced by growth factors removal and feeding with N2 medium with 200 μM ascorbic acid for 6–8 days. This protocol induces preferential differentiation of unmodified mouse ESC to DaNs (20–30% of the differentiated neurons), although lower proportions of serotoninergic (5–10%) are also present (Lee et al., 2000; Kim et al., 2002). Interestingly, DaNs, serotoninergic, and GABAergic neurons are present after grafting in Parkinsonian rats: DaNs represent 20% of surviving neurons, whereas serotonin^+^ cells are close to 5%, and cells positive to the GABAergic marker GAD67 are less than 2%, after 4–8 weeks (Kim et al., 2002).

### Enzyme Linked ImmunoSorbent Assay for GDNF

To measure secreted GDNF, 24 h-conditioned media were obtained from CTRL-ESC and GDNF-ESC during all five stages of DA differentiation. GDNF Emax Immunoassay system kit was purchased from Promega and was used following the manufacturer’s booklet.

### 6-OHDA Toxicity Assay *in vitro*

DA neurons were incubated with 200 μM 6-OHDA (Sigma) for 2 h and incubated at 37°C. Medium was removed and fresh medium was added. Twenty-four hours later, cells were fixed by evaluation by immunocytochemistry. This neurotoxin is taken up by the DA Transporter and therefore does not affect other neurons present in the differentiated cultures.

### Immunocytochemistry

Cells were fixed with 4% paraformaldehyde, permeabilized, and blocked with 10% normal goat serum and 0.3% Triton X-100 in PBS. Primary antibodies were incubated overnight in 10% normal goat serum in PBS and were applied as follows: mouse anti-OCT3/4, 1:1,000 (BD Biosciences Pharmingen, United States); rabbit anti-SOX2, 1:500 (R&D); mouse anti-β TUBULIN III (TUJ1), 1:1,000 (Covance); rabbit anti-Tyrosine Hydroxylase (TH) antibody, 1:1,000 (Pel-Freez, United States); rabbit anti-FOXA2, 1:500 (Millipore); rabbit anti-LMX1B, 1:200 (Abcam); rabbit anti-Serotonin (Sigma). Appropriate fluorescently labeled secondary antibodies were used alone or in combination, and nuclear detection with Hoechst 33258 1 μg/ml (Sigma, United States) is presented in some cases. For quantification of dopaminergic differentiation efficiency, the number of TH-positive somata were divided by the total number of neurons labeled by the TUJ1 antibodies and multiplied by 100. To further asses DaNs differentiation, we performed an analysis of area with co-localization of TUJ1 and TH signals with the FIJI program, using the JACoP package to obtain the percentage of colocalizing signals, as previously reported (Bolte and Cordelières, 2006; Dunn et al., 2011). Area was set as pixel intensity. The TUJ1 signal was established as the total area and the TH signal was set as the variable. Thus, the % of TH area was normalized using total TUJ1 area. For serotonin neurons, the percentage of neurons labeled with antibodies were normalized by the total number of nuclei. Fluorescent signals were detected using a Nikon A1R HD25 confocal microscope to detect Alexa 488, Alexa 568, Alexa 647, and Hoechst 33258 by exciting with different lasers.

### RT-qPCR

RNA was isolated using RNeasy Mini Kit (QIAGEN) and manufacturer’s instructions were followed. Complementary DNA (cDNA) was synthesized by SuperScript II Reverse Transcriptase (Thermo Fisher Scientific) from 2 μg of total RNA and used for RT-PCR amplification (Taq DNA Polymerase, Thermo Fisher Scientific). Amplification was performed with QuantiFAst SYBR Green PCR Master Mix (QIAGEN) and a Real-Time PCR Detection System (CFX96; Bio-Rad). Gene expression levels were determined using the following primers: *Oct4*: Fwd, TTG GGC TAG AGA AGG ATG TGG TT; Rev, GGA AAA GGG ACT GAG TAG AGT GTG G. For *Sox2*: Fwd, GCA CAT GAA CGG CTG GAG CAA CG; Rev, TGC TGC GAG TAG GAC ATG CTG TAG G. For *Th*: Fwd, CCA CTG GAG GCT GTG GTA TT; Rev, CCG GGT CTC TAA GTG GTG AA. For *Foxa2*: Fwd, CAG AAA AAG GCC TGA GGT G; Rev, CAG CAT ACT TTA ACT CGC TG. For *Lmx1b*: For *Gapdh*: Fwd, CAT CAC TGC CAC CCA GAA GAC TG; Rev, ATG CCA GTG AGC TTC CCG TTC AG.

### Animals

Female Wistar rats weighing 220–250 g were housed at 12 h light-dark cycle with food and water *ad libitum* at 22 ± 2°C. Surgical procedures were approved by the Instituto de Fisiología Celular-UNAM Animal Care and Use Committee (Protocols IV-68-15 and IV-152-19) and compiled local (NOM-062-ZOO-1999) and international guidelines.

### 6-OHDA Lesion, Apomorphine-Induced Rotational Test, and Non-pharmacological Behavioral Test

6-OHDA (Sigma) lesions were performed as previously described (Ungerstedt, 1968; Díaz-Martínez et al., 2013). This animal model shows behavioral and biochemical improvements after DaNs grafting (Kim et al., 2002; Rodríguez-Gómez et al., 2007) and therefore, we focused our analysis to this type of neurons. Rats were placed in an airtight anesthesia chamber supplied with 3% sevoflurane (Abbot Laboratories) in 95% O_2_–5% CO_2_ gas mixture. To minimize stress, rats were minimally handled and maintained with inhaled anesthetic (0.5–1.5% sevoflurane). Next, rats were injected with 12 μg of 6-OHDA in the left medial forebrain bundle with the following stereotactic coordinates in relation to bregma: antero-posterior (AP), −1.0 mm; lateral (L), 1.5 mm; and dorso-ventral (DV), −8.0 mm. The injection was performed over 4 min using a 30G needle. Thereafter, rats were allowed to recover for 30 days before a pharmacological test was performed. Apomorphine (1 mg/kg)-induced rotations were quantified over 60 min, and animals were classified as lesioned when they had more than 360 contralateral turns per hour. To assess motor behavior in the absence of pharmacological stimulation, we conducted the following test:

*Adjusting step test:* This evaluation was performed as described before (Kim et al., 2002; Díaz-Martínez et al., 2013). The rats were held with one hand, holding and lifting the hindlimbs and securing one of the forelimbs, and then moved in a forward direction over a flat surface for 0.9 m. Each forelimb was independently evaluated by counting the number of steps onto the surface while moving the animal. The value of the lesioned forelimb was normalized with the number of steps registered for the non-lesion value for each group. This evaluation was made blinded to the experimental treatment of animals.

### Grafting of DA Neurons Differentiated From CTRL-ESC and GDNF-ESC

All animals were immunosuppressed daily with cyclosporine A (10 mg/kg; GelPharma) starting 24 h before grafting. Hemiparkinsonian rats were grafted in the dorsal striatum with CTRL-ESC or GDNF-ESC derived TH-positive neurons differentiated as described before. DaNs from both cell lines were dissociated at days 2–3 of stage 5 and resuspended at a density of 0.5 × 10^6^ viable cells in 3 μl. Cells were grafted into the lesioned dorsolateral striatum (*n* = 7 for CTRL-ESC and GDNF-ESC) with the following coordinates: AP, 0.0 mm; L, 3.0 mm; DV, four deposits separated by 0.5 mm (from −5.5 mm to −4.0 mm). In sham animals (*n* = 8), 3 μl of N2 medium was injected with the same coordinates. Each animal was evaluated every 2 weeks for apomorphine-induced rotations, for 14 weeks.

### DA Quantification

Microdialysis experiments were performed at 14 weeks after grafting to measure DA release in the dorsal striatal region of animals grafted in the striatum with CTRL-ESC or GDNF-ESC and with sham surgery as described (Rodríguez-Gómez et al., 2007; Díaz-Martínez et al., 2013). *In vitro* recovery experiments with the dialysis membranes had values of 15–20% for DA and 3–4, dihydroxyphenylacetic acid (DOPAC). Probes were perfused with artificial cerebrospinal fluid at 2 μl per min for 1 h for tissue stabilization and fractions were collected every 12 min. Chemical depolarization with isosmotic medium (100 mM potassium chloride) was induced through the probe in fraction 4, to stimulate DA release; reversal of DA uptake was performed in fraction 9 by perfusion with 30 μM amphetamine. Monoamines were stabilized by adding 0.1 N perchloric acid, 0.02% EDTA, and 1% ethanol to the collection tubes. Quantification was made by HPLC, using a reversed-phase column (dC18, 3 μm; 2.1 mm × 50 mm; Atlantis, Waters) coupled to a precolumn (Nova-Pack Waters) with a mobile phase solution containing EDTA, 0.054 mM; citric acid, 50 mM; and octasulfonic acid, 0.1 mM dissolved in milli-Q water and mixed with methanol in a proportion of 97:3, respectively (pH = 2.9; flow rate = 0.35 ml/min). DA and DOPAC detection were performed by a single-channel electrochemical amperometric detector (Waters model 2465) at 450 mV with a temperature of 30°C, and quantified by peak height measurements against standard solutions. Resulting concentrations were not corrected by probe recovery.

### Stereological Counting

Animals were perfused with 0.9% saline solution and then with 4% paraformaldehyde in PBS. Brains were recovered and cryo-protected subsequently with 10%, 20%, and 30% sucrose. Slices of 30 μm were obtained in a cryostat and immunostained with anti-TH and anti-Tuj1 antibodies for DA neuron counting. Every 5th section was quantified, and the total number of DA neurons was calculated by multiplying the number of TH^+^ neurons per slice by the number of slices that contained grafted cells (Coggeshall and Lekan, 1996; Kim et al., 2002). We calculated the mean of the area covered by TUJ1^+^ cells per mm^3^ for at least 12 slices. To calculate the percentage of TH in each slice, the number of DaNs was normalized by the number of TUJ1^+^ cells.

### Statistical Analysis

Results are expressed as mean ± SEM. All experiments were performed at least in duplicate. Statistical differences were identified by one-way ANOVA or *t*-student test. Multiple comparisons were made by two-way ANOVA followed by *post hoc* Dunn’s test. GraphPad Prism version 8.0 was used for calculation of probability values.

## Results

### GDNF Expression in ESCs During Dopaminergic Differentiation

**FIGURE 1:**
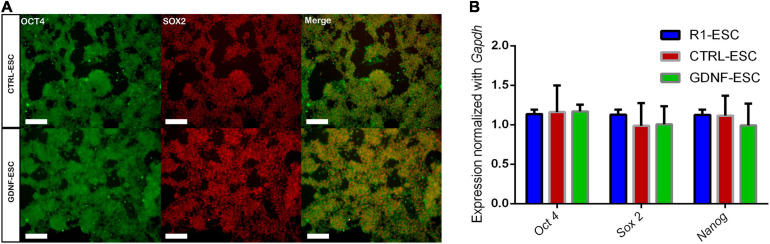
Pluripotency markers are unaffected in CTRL- and GDNF-ESC before differentiation. **(A)** Immunostaining for the pluripotency markers OCT4 and SOX2 in CTRL-ESC and GDNF-ESC, showing a high proportion of double-positive cells in both cell lines. Scale bar = 100 μm. Representative of five independent experiments. **(B)** RT-qPCR for the transcription factors *Oct4, Sox2*, and *Nanog* in both cell lines, normalized by *Gapdh* expression. R1 ESCs were used as a reference. No significant differences were observed; data from four independent experiments.

In order to address if transduction with the control vector or with the cDNA of hGDNF affects the pluripotent state of the generated lines of mESCs, cells were cultured for 48 h and we performed immunostaining for the pluripotency-associated markers OCT4 and SOX2. CTRL-ESC and GDNF-ESC presented similar proportions of double-positive cells (Figure 1A). NANOG was also analyzed with similar observations (data no shown). In agreement, no differences are detected in the expression of mRNA for *Oct4, Sox2*, and *Nanog* when comparing the parental R1 mESC with CTRL-ESC and GDNF-ESC by RT-qPCR (Figure 1B).

**FIGURE 2:**
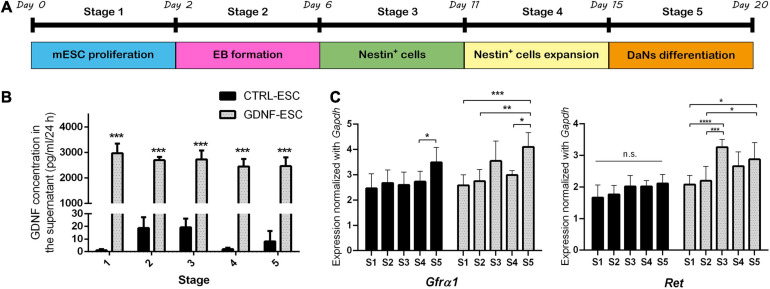
GDNF is released to the medium and its receptors are expressed during different stages of dopaminergic differentiation. **(A)** Scheme of five stages of the dopaminergic neuron differentiation protocol. **(B)** ELISA to quantify GDNF in 24 h-conditioned media from CTRL-ESC or GDNF-ESC during the five stages of differentiation. ***p < 0.001 vs. CTRL-ESC. **(C)** RT-qPCR showing mRNA levels of the GDNF receptors *Gfr*α*1* and *Ret* during differentiation of CTRL-ESC and GDNF-ESC. Values were normalized with *Gapdh* expression. *p < 0.05; **p < 0.01; ***p < 0.001; and ****p < 0.0001. *n* = 4 independent experiments.

After establishing that GDNF-ESC shows normal expression of pluripotency-related markers, we induced its differentiation to DaNs using a method previously described (Lee et al., 2000), which is a stepwise procedure involving five stages (Figure 2A). To investigate if GNDF expression and secretion can be detected during this differentiation protocol, conditioned media was collected from CTRL- and GDNF-ESC to quantify GDNF by enzyme linked immunosorbent assay (ELISA). GDNF concentration was significantly higher in GDNF-ESC when compared to CTRL-ESC at all stages of differentiation (Figure 2B). GDNF must bind to GFRα1 and Ret co-receptors to exert its effects. Therefore, we measured the expression of *Gfr*α*1* and *Ret* in the five stages of dopaminergic differentiation. No significant differences were observed between GDNF-ESC and CTRL-ESC (Figure 2C), but some differences were observed between stages, particularly at stage 5, where the expression of these transcripts was significantly increased compared to all the other stages in both cell lines. Together, these results indicate that GDNF is secreted and their receptors are expressed, suggesting that differentiation of ESCs to DaNs might be modified.

### GDNF-ESC Produce More DaNs That Are Resistant to 6-OHDA

**FIGURE 3:**
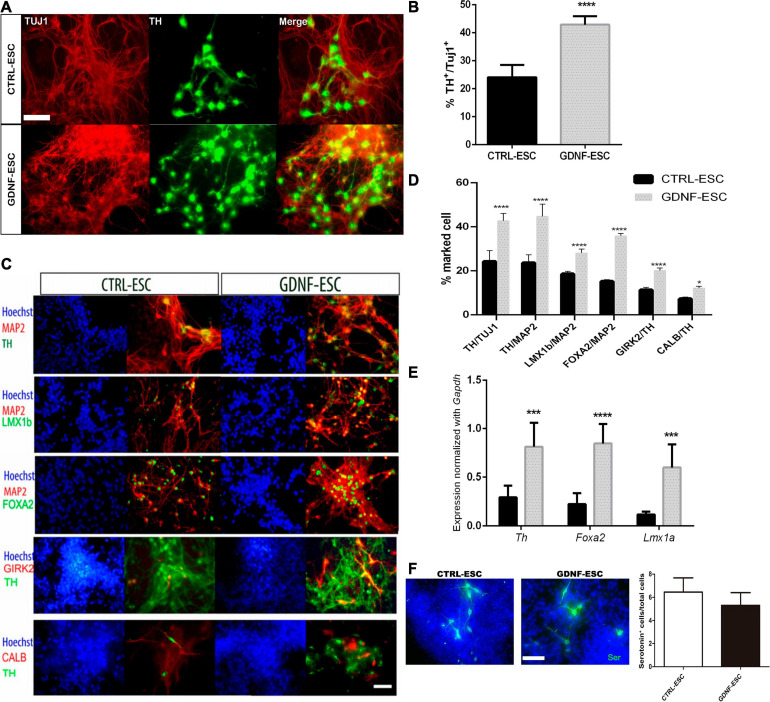
GDNF increases differentiation to DaNs. **(A)** Immunocytochemistry for TH and TUJ1 at 20 days of differentiation of GDNF-ESC or CTRL-ESC. **(B)** Quantification of DaNs, calculated as [total number of TH^+^ somas/total number of TUJ1^+^ somas] x100, to obtain percentage in cultures of 20 days showing a significant increase in TH-positive neurons in GDNF-ESC, compared to control. **(C)** Immunocytochemistry for ventral mesencephalic DaNs markers LMX1B, FOXA2, the A9 marker GIRK2 and Calbindin (CALB), combined with TH or Microtubule Associated Protein 2 (MAP2). **(D)** Quantification of these markers shows an increase that reached statistical significance for GDNF-ESC when quantified in four independent experiments. **(E)** Normalized mRNA expression of genes relevant for dopaminergic differentiation at day 20. Significant increases in expression levels in GDNF-ESC are observed, compared with CTRL-ESC. Data was normalized by *Gapdh* expression. **(F)** Immunocytochemistry and quantification of the percentage of Serotonin^+^ cells, related to the number of total cells, detected at day 20 of differentiation. Result from four independent experiments. *p < 0.05; ***p < 0.001; and ****p < 0.0001. Scale bar = 100 μm.

DaNs were differentiated from CTRL-ESC and GDNF-ESC. At stage 2, we observed that GDNF-ESC EBs were larger than those generated from CTRL-ESC. Quantification of the diameter showed significant differences (0.98 ± 0.02 μm, CTRL; 1.03 ± 0.03 μm, GDNF; *p* < 0.0001), suggesting that GDNF is increasing proliferation during EB formation, which is consistent with previous findings (Cortés et al., 2016). The final differentiation phase is stage 5, where neurons expressing β-III Tubulin, detected by the TUJ1 antibody, start to express TH, the limiting enzyme in DA production. Postmitotic neurons were evaluated for this marker: a significantly higher number of TH-positive cells were observed in GDNF-ESC compared with CTRL-ESC (Figures 3A,B). The increased dopaminergic differentiation in GDNF-ESC was confirmed by measuring the % of TH area, relative to TUJ1 labeling: CTRL-ESC = 21.6 ± 2.9%; GDNF-ESC = 36.8 ± 1.2%; significant difference after *t*-test for *n* = 6 (*p* < 0.01). Furthermore, expression of other markers of DaNs from the ventral mesencephalic area, like FOXA2, LMX1B, GIRK2, and Calbindin, were also significantly increased in GNDF-ESC (Figures 3C,D). This was complemented by detection of the dopaminergic cell markers *Th, Foxa2*, and *Lmx1a* through RT-qPCR (Figure 3E), showing that GDNF overexpression is increasing efficiency for differentiation into DaNs. This protocol also produces a low proportion of serotoninergic neurons, so we decide to quantify this neuronal population. The number of serotonin-positive neurons was not modified in GNDF-ESC when compared to CTRL-ESC (Figure 3F).

**FIGURE 4:**
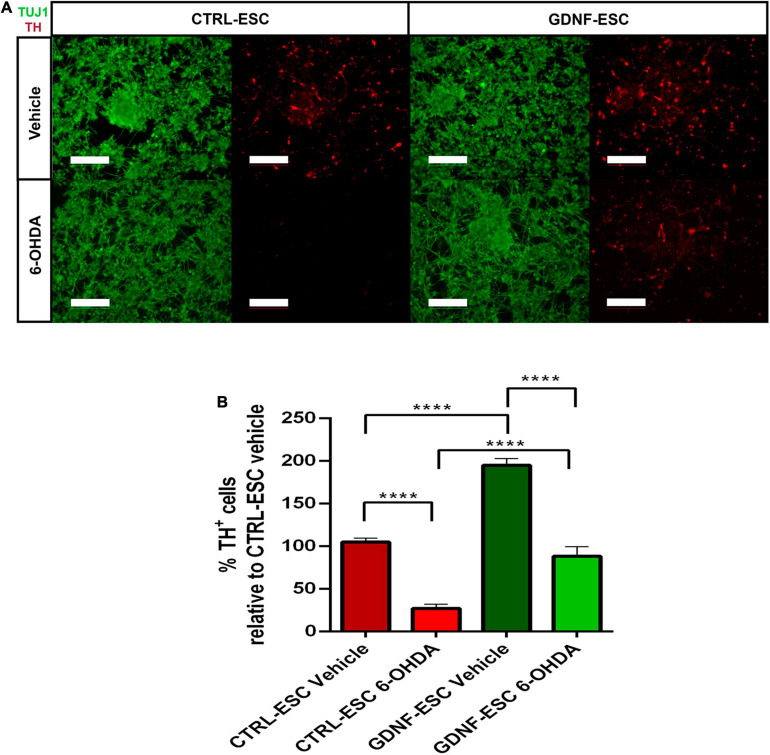
Over-expression of GDNF promotes survival of TH-positive neurons after a challenge with 6-OHDA *in vitro*. **(A)** Immunocytochemistry for TUJ1 and TH after vehicle incubation for 24 h (upper panels) and after 6-OHDA treatment (bottom panels). Note that GDNF-ESC has a higher proportion of DaNs than CTRL-ESC in vehicle and after 6-OHDA. **(B)** Quantification of the % of TH-positive cells in experiments at 20 days of differentiation, related to CTRL-ESC treated with vehicle, which was considered 100%. Results are from four independent experiments. ****p < 0.0001. Scale bar = 100 μm.

To test if transgenic GDNF has a neurotrophic effect on DaNs after a toxic challenge with the dopaminergic neurotoxin 6-OHDA, differentiated cultures of 20 days were treated with vehicle or 200 μM of 6-OHDA for 2 h, and assessed after 24 h. In CTRL-ESC, 6-OHDA caused a significant decrease: only 27% of TH-positive cells survived, relative to vehicle incubation. In contrast, GDNF-ESC cells were significantly less sensitive to this toxin, since 48% of TH+, relative to vehicle, were present (Figures 4A,B). The neuroprotection in DaNs differentiated from GDNF-ESC was also observed by the significant increase in the % of TH^+^ area, normalized by TUJ1: CTRL-ESC with 6-OHDA = 9.0 ± 1.5%; GDNF-ESC plus 6-OHDA = 54.2 ± 6.6%; *n* = 4, *p* < 0.0001. Together, these results show that transgenic GDNF increases the proportion of ventral mesencephalic neurons and confer resistance to 6-OHDA cytotoxic challenge.

### Transplantation of ESC-Derived DaNs in the Striatum Promotes Behavioral Recovery and DA Release in Hemiparkinsonian Rats

**FIGURE 5:**
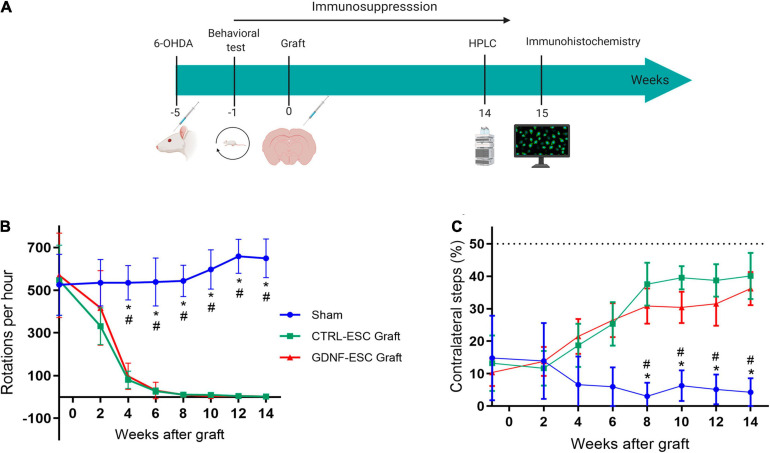
Behavioral evaluation of Hemiparkinsonian rats after grafting. **(A)** Scheme of the protocol for *in vivo* experiments. **(B)** Apomorphine-induced rotations in animals receiving sham, CTRL-ESC, or GDNF-ESC treatments and followed 14 weeks post-grafting. Statistical differences were observed between sham and both types of grafts. The initial values are pre-grafting. **(C)** Grafting of CTRL-ESC and GDNF-ESC caused a significant recovery of forelimb asymmetry in the stepping test, when compared to the sham group. The dotted line represents forelimb use in a non-lesioned animal. *p < 0.05 vs. CTRL-ESC; ^#^*p* < 0.05 vs. GDNF-ESC. The number of animals is eight for the sham group and seven for the grafted groups.

The functionality of *in vitro* differentiated DaNs can be assessed by intrastriatal transplantation in rats lesioned with 6-OHDA (Figure 5A). Animals were injected with this dopaminergic neurotoxin in the right medial forebrain bundle. A successful lesion was considered when animals presented an apomorphine-induced rotational asymmetry of >360 rotations per hour. This animal model (Ungerstedt and Arbuthnott, 1970) has been widely used to test behavioral alterations and, indirectly, restoration of DA levels. Hemiparkinsonian rats were grafted with 5 × 10^5^ cells from differentiating cultures (day 18) of CTRL-ESC or GDNF-ESC. We measured the behavioral recovery of Parkinsonian rats using the rotational and stepping tests. For rats receiving sham transplantation, the behavioral alterations caused by the lesion were present for 14 weeks post-surgery. Grafts of CTRL-ESC and GDNF-ESC showed a significant recovery in apomorphine-induced rotations (Figure 5B) lasting 14 weeks post-transplantation. A similar correction of forelimb asymmetry in the stepping test was found in rats grafted with CTRL-ESC and GDNF-ESC (Figure 5C).

**FIGURE 6:**
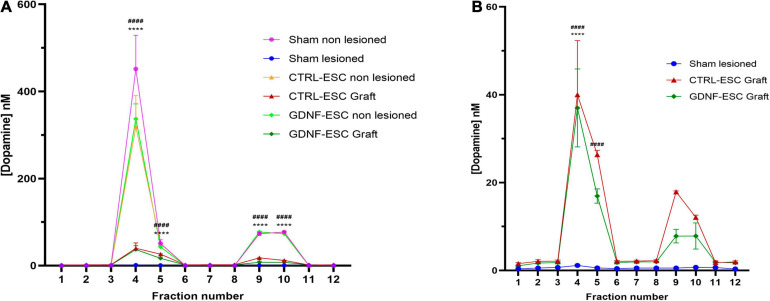
Quantification of DA release *in vivo* in lesioned animals grafted with CTRL-ESC or GDNF-ESC at 14 weeks post-grafting. **(A)** DA concentrations measured by microdialysis and HPLC detection in basal conditions (fractions 1–3), after 100 mM KCl isosmotic medium (fraction 4), and 30 μM amphetamine (fraction 9) in the lesioned and non-lesioned sides of sham and grafted animals. **(B)** Amplification of DA levels shown in panel **(A)** for lesioned sides in grafted CTRL-ESC, GDNF-ESC, and sham groups; *n* = 5; ****p < 0.0001 vs. fraction 3 or fraction 8 in CTRL-ESC and ^####^*p* < 0.0001 vs. fraction 3 or 8 in GDNF-ESC.

To correlate behavioral improvements with DA release in grafted rats, extracellular DA levels were measured in the striatum by microdialysis following two pharmacological challenges applied through the cannula: (a) depolarization induced by K^+^ ions (isosmotic medium with 100 mM KCl) and (b) administration of 30 μM amphetamine, which causes the release of DA *via* the DA Transporter. As a control, the non-lesioned striatum of all animals showed a significant potassium-stimulated DA release, compared with basal levels, and a significant DA accumulation after amphetamine application through the dialysis membrane. Intrastriatal grafting of DaNs from CTRL-ESC and GDNF-ESC causes depolarization- and amphetamine-induced DA release in the striatum *in vivo* to a similar extent, in agreement with behavioral data. In contrast, the lesioned side from sham rats did not present significant DA increases (Figures 6A,B).

### Distribution and Engraftment of GDNF-ESC and CTRL-ESC Following Transplantation

**FIGURE 7:**
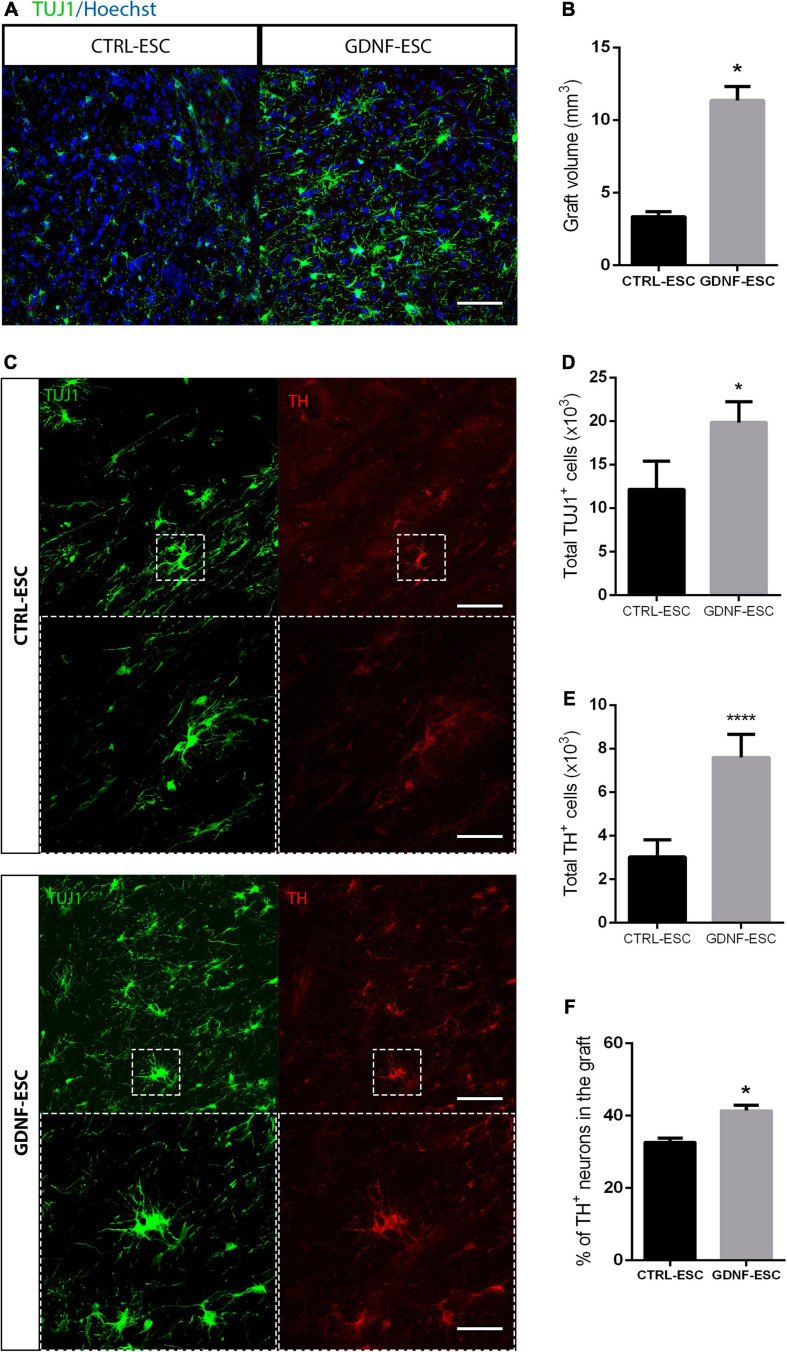
Grafting of GDNF-ESC-derived DaNs into the striatum of lesioned animals results in higher numbers of DaNs, compared to CTRL-ESC. **(A)** Immunostaining for TUJ1 in the striatum of grafted animals at 15 weeks post-graft. Scale bar = 100 mm. **(B)** Quantification of graft volume measured after TUJ1 staining in 12 slices for both grafted groups. **(C)** Immunohistochemistry for TUJ1 and TH in the striatum of grafted animals. GDNF-ESC grafts have increased numbers of TH^+^ cells. Scale bar = 100 mm. The bottom panels show the amplification of the doted square. Scale bar = 35 mm. **(D)** Quantification of total TUJ1^+^ cells in CTRL-ESC and GDNF-ESC grafts. **(E)** Quantification of total TH^+^ cells in grafts with CTRL-ESC or GDNF-ESC. **(F)** Quantification of the percentage of TH-positive neurons in the striatum of grafted animals, calculated as follows: [total number of TH^+^ somas/total number of TUJ1^+^ somas] ×100. Results from 7 CTRL-ESC and 7 GDNF-ESC grafted animals. *p < 0.05 and ****p < 0.0001.

To test if GDNF is capable of promoting survival, we analyzed the number of DaNs in the striatum of grafted animals. Both CTRL-ESC and GNDF-ESC showed engraftment and survival of TH^+^ cells in the striatum of transplanted animals, 14 weeks after grafting. Both types of grafts presented TH^+^ processes into the striatum. In the non-lesioned sides of all groups, TH^+^ innervation from the DaNs of the substantia nigra were found, as expected; this innervation was absent in the lesioned side in the sham animals. Interestingly, GDNF-ESC graft size was significantly increased compared to CTRL-ESC (Figures 7A,B) and the number of total TUJ1^+^ was increased, too (Figures 7C,D). The number of DaNs was higher in animals grafted with GDNF-ESC (Figures 7C,E). Furthermore, the percentage of cells expressing the dopaminergic marker TH in grafts of GDNF-ESC-derived DaNs was significantly higher compared with DaNs from CTRL-ESC (Figures 7C,F), indicating that GDNF can promote DaNs survival after grafting. This result was further confirmed by measuring the % of TH area in both groups: CTRL-ESC = 16.2 ± 0.8% vs. GDNF-ESC = 22.7 ± 1.0 (*p* < 0.0001 after comparison with *t*-test, *n* = 7).

## Discussion

We describe a positive effect of continuous GDNF secretion on midbrain dopaminergic differentiation and also an autocrine neurotrophic effect when transgenic DaNs are exposed to 6-OHDA. GDNF-ESC-derived DaNs were grafted in Parkinsonian rats and induced behavioral recovery as well as dopamine release in the striatum, similar to CTRL-ESC-derived DaNs, but with increased numbers of surviving DaNs, indicating that the continuous release of GDNF does not cause undesired effects and promotes DaNs survival after grafting.

In the current study, we have characterized mESCs that constitutively express and release GDNF, a strong neurotrophic factor that promotes survival and maturation of DaNs (Lin et al., 1993; Sauer et al., 1995; Åkerud et al., 2001). This GDNF-secreting cell line differentiated more efficiently to DaNs compared to CTRL-ESC. Although these ESCs express receptors for this neurotrophic factor, in our experiments, pluripotency-associated factors such as Oct4, Sox2, and Nanog were not altered, suggesting that cells remain pluripotent. It is well known that GDNF is a strong inducer of proliferation and differentiation of enteric nervous system and parasympathetic ganglia precursors *in vivo* (Enomoto et al., 2000; Rossi et al., 2000; Airaksinen and Saarma, 2002; Gianino, 2003; Cortés et al., 2017); however, its role in early differentiation of ESCs to DaNs *in vitro* is still unclear. Interestingly, during EB formation the size of these aggregates increased significantly in GDNF-ESC; furthermore, the number of Nestin^+^ cells migrating away from the EBs, during the first 2 days of stage 3, was significantly increased in ESC-GDNF (data not shown), suggesting that GDNF influence the proliferation and migration of neural precursors. This is consistent with previous work reporting that human recombinant GNDF can increase the proliferation rate, the size of EBs, and spinal motor neuron differentiation form pluripotent stem cells (Lamas et al., 2014; Cortés et al., 2016). The cell population that is responsive to GDNF in EBs, and the mechanisms triggered by this trophic factor, remain as an open question, requiring further investigations. Interestingly, the effect of recombinant GDNF on control ESC differentiated to motor neurons was abolished by the addition of anti-GDNF antibodies. The conditions of exogenous addition of recombinant GDNF would be mimicked by the addition of conditioned medium from GDNF-ESC to differentiating CTRL-ESC to test if an increase of DaNs is observed, which would be an interesting follow-up experiment.

Most of the protocols for the differentiation of pluripotent stem cells to dopaminergic lineages include GNDF as part of the supplementation of the medium, especially at late stages to increase the survival of TH^+^ cells (Lee et al., 2000; Kriks et al., 2011; Kirkeby et al., 2017). In this work cells expressed GDNF during all the differentiation protocols and we observed an increase in the number of TH^+^ cells vs. CTRL-ESC. Moreover, other mesencephalic markers were increased, such as Lmx1b, Foxa2. In the midbrain there are two dopaminergic populations: neurons from ventral tegmental area (VTA) express Calbindin and those from the SNpc are positive for Girk2, which is a G-protein-regulated inward-rectifier potassium channel (Inanobe et al., 1999; Neuhoff et al., 2002; Roeper, 2013). We assessed the proportion of Calbindin- and GIRK2-positive cells and found significant increases in both dopaminergic cell types. As previously reported, GDNF can provide survival signals to different niches of neurons such as sympathetic, enteric, motor, and other catecholaminergic neurons such as noradrenergic (Lin et al., 1993; Coulpier and Ibáñez, 2004; Pascual et al., 2008; Cortés et al., 2016; Ito and Enomoto, 2016; Enterría-Morales et al., 2020b, a). Interestingly, the proportion of differentiated serotonin neurons was unchanged in GDNF-ESC. This suggests that GDNF might increase the number of DaNs derived from ESC by promoting cell survival but also by having an effect as an inductor of dopaminergic differentiation.

DaNs differentiated from GDNF-ESC were more resistant to the cytotoxic challenge with 6-OHDA *in vitro*, as expected given the well-described neuroprotective effect of GDNF (Lin et al., 1993; Sauer et al., 1995; Kramer and Liss, 2015; Meka et al., 2015). However, this effect has only been observed when exogenous GDNF is added to the medium (Meyer et al., 2001) or when DaNs are co-cultured with cells that secrete GDNF (Åkerud et al., 2001), but not when the same cell population is producing GDNF. This type of system could generate an autocrine effect in differentiated DaNs, leading to increased cell survival *in vitro* or *in vivo*, especially after grafting in animal models of PD (Zurn et al., 2001). In this manner, additional allogenic transplants or viral methods for delivery of GDNF, such as AAV2 systems, can be avoided (Kells et al., 2012; Tenenbaum and Humbert-Claude, 2017). Recent works have shown that co-grafting of DaNs with systems that in a constitutively (Gantner et al., 2020) or regulated way (Perez-Bouza et al., 2017; Widmer, 2018) secretes GDNF, can promote behavioral recovery in animal models of PD. However, as mentioned before, none of these systems express the GDNF by the grafted cells and require another cell type to express this neurotrophic factor, such as mesenchymal stem cells, myoblasts, or by direct infusion of GDNF (Yurek, 1998; Wyse et al., 2014).

The loss of striatal dopamine results in motor dysfunction, including resting tremor, muscular rigidity, bradykinesia, and postural instability in PD patients (Dauer and Przedborski, 2003). Currently, pharmacologic DA supplementation is the most used strategy to reduce the symptoms of PD. Transplantation of DaNs has widely been used in laboratory animal models and in several clinical trials (Kordower et al., 1995; Piccini et al., 1999; Kordower et al., 2017). The most popular procedures have involved ectopic transplantation of healthy DaNs into the striatum, but this technique has proven unsatisfactory in clinical trials for multiple reasons, one of them the number of survival neurons after the graft (Björklund and Lindvall, 2000; Brundin et al., 2010). For this reason, new strategies have emerged focusing on preventing the progressive loss of neurons at early stages of the disease using neurotrophic factors (Axelsen and Woldbye, 2018; Nasrolahi et al., 2018; Gouel et al., 2019; Wang et al., 2020). However, clinical trials of GDNF have shown that this therapy has serious limitations such as intraputaminal delivery and the poor capacity of diffusion, added to the fact that there was no motor improvement in the patients (Whone et al., 2019a, b). Thus, in this work, we used both cell replacement and GDNF delivery to test enhanced survival of grafted DaNs in Hemiparkinsonian rats. It is well known that injection of 6-OHDA results in a severe and acute loss of SNpc DaNs and causes behavioral deficits measured by apomorphine-induced rotations (Ungerstedt, 1968; Ungerstedt and Arbuthnott, 1970). Our study shows that grafts of ESC-derived DaNs that express constitutively GNDF can promote behavioral recovery in animals that were previously lesioned with 6-OHDA in both pharmacological and non-pharmacological tests. Interestingly, no difference in recovery time was observed between control cell and GDNF-expressing cells. As previously reported, consistent behavioral recovery has been reported after grafting of ESC-derived DaNs in the dorsal striatum (Kim et al., 2002; Kriks et al., 2011; Díaz-Martínez et al., 2013; Kirkeby et al., 2017; Gantner et al., 2020) 4 weeks after transplantation of mouse ESC-derived DaNs, and 12 weeks using human ESC-derived DaNs. Interestingly, continuous GDNF presence in the graft did not result in uncontrolled growth since the aspect of grafts for both cell types studied here is similar, although the volume was increased in GDNF-ESC.

GDNF is a strong regulator of excitability in DaNs (Yang et al., 2001) and it has been demonstrated that human DaNs differentiated from ESCs are capable of regulating DA synthesis when GDNF is delivered and released at multiple times (Gantner et al., 2020). Grafts of DaNs differentiated from mESCs or human ESCs can increase the levels of DA in the striatum of transplanted animals without GDNF over-expression (Piccini et al., 1999; Rodríguez-Gómez et al., 2007; Díaz-Martínez et al., 2013). Dopamine levels in the lesioned striatum of grafted animals were higher in comparison with sham animals, but no difference between CTRL-ESC and GDNF-ESC was observed. This is in contrast with previous reports that demonstrate that GDNF can increase dopamine levels *via* the regulation of *TH* gene expression in a *Ret*-dependent response in human neuroblastoma cell lines (Xiao et al., 2002) and in grafts of human ESC-derived DaNs into an environment of GDNF overexpression (Gantner et al., 2020). However, in other studies, sustained GDNF expression induces down-regulation of DA levels, as well as of DAT activity, without altering *TH* mRNA levels as a compensatory mechanism and, thus, reduces dopaminergic function (Georgievska et al., 2002; Yang et al., 2009; Barroso-Chinea et al., 2016; Chtarto et al., 2016; Tenenbaum and Humbert-Claude, 2017). For these reasons, GDNF concentration and expression should ideally be regulated in future studies to avoid unwanted compensatory mechanisms and prevent the downregulation of DA release in grafts of ESC-derived DaNs.

We demonstrate that the sustained expression of GDNF increases the number of TH-positive neurons after grafting of ESC-derived DaNs. Unfortunately, this increased number did not improve the time of recovery nor increased DA release in the brain, showing that sustained expression of GDNF does not alter the beneficial effects of grafted DaNs. It has been proposed that integration and survival in the host tissue of DaNs are key features to improve functional recovery (Brundin et al., 2000; Karlsson et al., 2000); however, our results show that both grafted groups had similar recovery in pharmacological and no pharmacological tests, suggesting that the number of DaNs in the grafts of CTRL-ESC were sufficient to induce behavioral and neurochemical improvements. Recent works have proposed that cell survival induced by GDNF is not necessarily the main key to improve motor symptoms, but increased fiber growth or axonal sprouting (Clavreul et al., 2006; Grealish et al., 2014; Perez-Bouza et al., 2017). Furthermore, it seems that the time in which GDNF is present relative to grafting determines how efficient the axonal sprouting and the improvement in non-pharmacological tests will be (Gantner et al., 2020). This could be associated to the “candy-store” effect (Santosa et al., 2013; Marquardt et al., 2015) where the axons only grow to the major concentration of GDNF.

In summary, we provide evidence for the following: (1) Transgenic GDNF does not affect pluripotency of mESCs; (2) The positive effects of sustained GDNF release on dopaminergic differentiation *in vitro*; (3) GDNF-ESC are less sensitive to the toxic action of 6-OHDA; (4) After grafting, both CTRL- and GDNF-ESC induced behavioral recovery and DA release in the brain of lesioned animals; (5) GDNF in genetically modified mESC-derived DaNs increased the number of surviving DaNs in the brains of a rodent model of PD. Although CTRL-ESC can promote behavioral recovery in lesioned animals after grafting, the number of TH-positive neurons is lower than in GDNF-ESC grafts. This difference in the number of TH^+^ cells has no effect in pharmacological and non-pharmacological tests or neither elevating DA release measured by the HPLC. Our findings suggest that a better strategy to deliver GDNF in grafts should be considered, such as a regulable expression system activated by tetracycline, which proved to be effective in stem cells (Marquardt et al., 2015; Das et al., 2016; Guo et al., 2017; Bara et al., 2018; Ge et al., 2018).

## Data Availability Statement

The raw data supporting the conclusions of this article will be made available by the authors, without undue reservation.

## Ethics Statement

The animal study was reviewed and approved by Instituto de Fisiología Celular-UNAM Animal Care and Use Committee (Protocols IV-68-15 and IV-152-19) and compiled local (NOM-062-ZOO-1999) and international guidelines.

## Author Contributions

RL-R: conception and design, collection and assembly of data, analysis and interpretation, manuscript writing, and final approval of manuscript. DC, AL-O, KS, FC, LR, and EE: collection and assembly of data and final approval of manuscript. IV: financial support, data analysis and interpretation, manuscript writing, and final approval of manuscript. All authors contributed to the article and approved the submitted version.

## Conflict of Interest

The authors declare that the research was conducted in the absence of any commercial or financial relationships that could be construed as a potential conflict of interest.

## References

[B1] Airaksinen M. S. Saarma M. (2002). The GDNF family: signalling, biological functions and therapeutic value. *Nat. Rev. Neurosci.* 3 383–394. 10.1038/nrn812 11988777

[B2] Åkerud P. Canals J. M. Snyder E. Y. Arenas E. (2001). Neuroprotection through delivery of glial cell line-derived neurotrophic factor by neural stem cells in a mouse model of Parkinson’s disease. *J. Neurosci.* 21 8108–8118. 10.1523/jneurosci.21-20-08108.2001 11588183 PMC6763865

[B3] Axelsen T. M. Woldbye D. P. D. (2018). Gene therapy for Parkinson’s disease, an update. *J. Parkinsons Dis.* 8 195–215. 10.3233/jpd-181331 29710735 PMC6027861

[B4] Bara J. J. Dresing I. Zeiter S. Anton M. Daculsi G. Eglin D. (2018). A doxycycline inducible, adenoviral bone morphogenetic protein-2 gene delivery system to bone. *J. Tissue Eng. Regen. Med.* 12 e106–e118. 10.1002/term.2393 27957814

[B5] Barroso-Chinea P. Cruz-Muros I. Afonso-Oramas D. Castro-Hernández J. Salas-Hernández J. Chtarto A. (2016). Long-term controlled GDNF over-expression reduces dopamine transporter activity without affecting tyrosine hydroxylase expression in the rat mesostriatal system. *Neurobiol. Dis.* 88 44–54. 10.1016/j.nbd.2016.01.002 26777664

[B6] Björklund A. Lindvall O. (2000). Cell replacement therapies for central nervous system disorders. *Nat. Neurosci.* 3 537–544. 10.1038/75705 10816308

[B7] Bolte S. Cordelières F. P. (2006). A guided tour into subcellular colocalization analysis in light microscopy. *J. Microsc.* 224 213–232. 10.1111/j.1365-2818.2006.01706.x 17210054

[B8] Brundin D. P. Karlsson J. Emgård M. Schierle G. S. K. Hansson O. Petersén Å, et al. (2000). Improving the survival of grafted dopaminergic neurons: a review over current approaches. *Cell Transplant.* 9 179–195. 10.1177/096368970000900205 10811392

[B9] Brundin P. Barker R. A. Parmar M. (2010). “Chapter 14 - Neural grafting in Parkinson’s disease: problems and possibilities,” in *Recent Advances in Parkinson’S Disease*, eds Björklund A. Angela Cenci M. (Amsterdam: Elsevier), 265–294. 10.1016/S0079-6123(10)84014-2 20887880

[B10] Cenci M. A. (2014). Presynaptic mechanisms of l-DOPA-induced dyskinesia: the findings, the debate, and the therapeutic implications. *Front. Neurol.* 5:242. 10.3389/fneur.2014.00242 25566170 PMC4266027

[B11] Chtarto A. Humbert-Claude M. Bockstael O. Das A. T. Boutry S. Breger L. S. (2016). A regulatable AAV vector mediating GDNF biological effects at clinically-approved sub-antimicrobial doxycycline doses. *Mol. Ther. Methods Clin. Dev.* 3:16027. 10.1038/mtm.2016.27 27069954 PMC4813607

[B12] Chung S. Sonntag K. C. Andersson T. Bjorklund L. M. Park J. J. Kim D. W. (2002). Genetic engineering of mouse embryonic stem cells by Nurr1 enhances differentiation and maturation into dopaminergic neurons. *Eur. J. Neurosci.* 16 1829–1838. 10.1046/j.1460-9568.2002.02255.x 12453046 PMC2610444

[B13] Clavreul A. Sindji L. Aubert-Pouëssel A. Benoît J.-P. Menei P. Montero-Menei C. N. (2006). Effect of GDNF-releasing biodegradable microspheres on the function and the survival of intrastriatal fetal ventral mesencephalic cell grafts. *Eur. J. Pharm. Biopharm.* 63 221–228. 10.1016/j.ejpb.2005.11.006 16497494

[B14] Coggeshall R. E. Lekan H. A. (1996). Methods for determining numbers of cells and synapses: a case for more uniform standards of review. *J. Comp. Neurol.* 364 6–15. 10.1002/(SICI)1096-9861(19960101)364:1<6::AID-CNE2>3.0.CO;2-9 8789272

[B15] Collier T. J. Sortwell C. E. Daley B. F. (1999). Diminished viability, growth, and behavioral efficacy of fetal dopamine neuron grafts in aging rats with long-term dopamine depletion: an argument for neurotrophic supplementation. *J. Neurosci.* 19 5563–5573. 10.1523/JNEUROSCI.19-13-05563.1999 10377363 PMC6782306

[B16] Cortés D. Carballo-Molina O. A. Castellanos-Montiel M. J. Velasco I. (2017). The non-survival effects of glial cell line-derived neurotrophic factor on neural cells. *Front. Mol. Neurosci.* 10:258. 10.3389/fnmol.2017.00258 28878618 PMC5572274

[B17] Cortés D. Robledo-Arratia Y. Hernández-Martínez R. Escobedo-Ávila I. Bargas J. Velasco I. (2016). Transgenic GDNF positively influences proliferation, differentiation, maturation and survival of motor neurons produced from mouse embryonic stem cells. *Front. Cell. Neurosci.* 10:217. 10.3389/fncel.2016.00217 27672361 PMC5018488

[B18] Coulpier M. Ibáñez C. F. (2004). Retrograde propagation of GDNF-mediated signals in sympathetic neurons. *Mol. Cell. Neurosci.* 27 132–139. 10.1016/j.mcn.2004.06.001 15485769

[B19] Das A. Tenenbaum L. Berkhout B. (2016). Tet-on systems for doxycycline-inducible gene expression. *Curr. Gene Ther.* 16 156–167. 10.2174/1566523216666160524144041 27216914 PMC5070417

[B20] Dauer W. Przedborski S. (2003). Parkinson’s disease. *Neuron* 39 889–909. 10.1016/S0896-6273(03)00568-3 12971891

[B21] Deng X. Liang Y. Lu H. Yang Z. Liu R. Wang J. (2013). Co-Transplantation of GDNF-overexpressing neural stem cells and fetal dopaminergic neurons mitigates motor symptoms in a rat model of Parkinson’s disease. *PLoS One* 8:e80880. 10.1371/journal.pone.0080880 24312503 PMC3849044

[B22] Díaz-Martínez N. E. Tamariz E. Díaz N. F. García-Peña C. M. Varela-Echavarría A. Velasco I. (2013). Recovery from experimental Parkinsonism by semaphorin-guided axonal growth of grafted dopamine neurons. *Mol. Ther.* 21 1579–1591. 10.1038/mt.2013.78 23732989 PMC3734661

[B23] Dunn K. W. Kamocka M. M. McDonald J. H. (2011). A practical guide to evaluating colocalization in biological microscopy. *Am. J. Physiol. Cell Physiol.* 300 C723–C742. 10.1152/ajpcell.00462.2010 21209361 PMC3074624

[B24] Enomoto H. Heuckeroth R. O. Golden J. P. Johnson E. M. Milbrandt J. (2000). Development of cranial parasympathetic ganglia requires sequential actions of GDNF and neurturin. *Development* 127 4877–4889. 11044402 10.1242/dev.127.22.4877

[B25] Enterría-Morales D. Del Rey N. L.-G. Blesa J. López-López I. Gallet S. Prévot V. (2020a). Molecular targets for endogenous glial cell line-derived neurotrophic factor modulation in striatal parvalbumin interneurons. *Brain Commun.* 2:fcaa105. 10.1093/braincomms/fcaa105 32954345 PMC7472905

[B26] Enterría-Morales D. López-López I. López-Barneo J. de Tassigny X. (2020b). Role of glial cell line-derived neurotrophic factor in the maintenance of adult mesencephalic catecholaminergic neurons. *Mov. Disord.* 35 565–576. 10.1002/mds.27986 31930748

[B27] Freed C. R. Greene P. E. Breeze R. E. Tsai W. Y. DuMouchel W. Kao R. (2001). Transplantation of embryonic dopamine neurons for severe Parkinson’s disease. *N. Engl. J. Med.* 344 710–719. 10.1056/NEJM200103083441002 11236774

[B28] Gantner C. W. Luzy I. R. de, Kauhausen J. A. Moriarty N. Niclis J. C. Bye C. R. (2020). Viral delivery of GDNF promotes functional integration of human stem cell grafts in Parkinson’s disease. *Cell Stem Cell* 26 511–526.e5. 10.1016/j.stem.2020.01.010 32059808

[B29] Ge G. Chen C. Guderyon M. J. Liu J. He Z. Yu Y. (2018). Regulatable lentiviral hematopoietic stem cell gene therapy in a mouse model of Parkinson’s disease. *Stem Cells Dev.* 27 995–1005. 10.1089/scd.2018.0030 29562865

[B30] Georgievska B. Kirik D. Björklund A. (2002). Aberrant sprouting and downregulation of tyrosine hydroxylase in lesioned nigrostriatal dopamine neurons induced by long-lasting overexpression of glial cell line derived neurotrophic factor in the striatum by lentiviral gene transfer. *Exp. Neurol.* 177 461–474. 10.1006/exnr.2002.8006 12429192

[B31] Gianino S. (2003). GDNF availability determines enteric neuron number by controlling precursor proliferation. *Development* 130 2187–2198. 10.1242/dev.00433 12668632

[B32] Gouel F. Rolland A. S. Devedjian J. C. Burnouf T. Devos D. (2019). Past and future of neurotrophic growth factors therapies in ALS: from single neurotrophic growth factor to stem cells and human platelet lysates. *Front. Neurol.* 10:835. 10.3389/fneur.2019.00835 31428042 PMC6688198

[B33] Grealish S. Diguet E. Kirkeby A. Mattsson B. Heuer A. Bramoulle Y. (2014). Human ESC-derived dopamine neurons show similar preclinical efficacy and potency to fetal neurons when grafted in a rat model of Parkinson’s disease. *Cell Stem Cell* 15 653–665. 10.1016/j.stem.2014.09.017 25517469 PMC4232736

[B34] Guo J. Ma D. Huang R. Ming J. Ye M. Kee K. (2017). An inducible CRISPR-ON system for controllable gene activation in human pluripotent stem cells. *Protein Cell* 8 379–393. 10.1007/s13238-016-0360-8 28116670 PMC5413595

[B35] Hefti F. Melamed E. Wurtman R. J. (1981). The site of dopamine formation in rat striatum after L-dopa administration. *J. Pharmacol. Exp. Ther.* 217 189–197. 7205652

[B36] Inanobe A. Yoshimoto Y. Horio Y. Morishige K. I. Hibino H. Matsumoto S. (1999). Characterization of G-protein-gated K+ channels composed of Kir3.2 subunits in dopaminergic neurons of the substantia nigra. *J. Neurosci.* 19 1006–1017. 10.1523/JNEUROSCI.19-03-01006.1999 9920664 PMC6782136

[B37] Ito K. Enomoto H. (2016). Retrograde transport of neurotrophic factor signaling: implications in neuronal development and pathogenesis. *J. Biochem.* 160 77–85. 10.1093/jb/mvw037 27318359

[B38] Karlsson J. Emgård M. Gidö G. Wieloch T. Brundin P. (2000). Increased survival of embryonic nigral neurons when grafted to hypothermic rats. *Neuroreport* 11 1665–1668. 10852221 10.1097/00001756-200006050-00014

[B39] Kearns C. M. Gash D. M. (1995). GDNF protects nigral dopamine neurons against 6-hydroxydopamine *in vivo*. *Brain Res.* 672 104–111. 10.1016/0006-8993(94)01366-P 7749731

[B40] Kells A. P. Forsayeth J. Bankiewicz K. S. (2012). Glial-derived neurotrophic factor gene transfer for Parkinson’s disease: anterograde distribution of AAV2 vectors in the primate brain. *Neurobiol. Dis.* 48 228–235. 10.1016/j.nbd.2011.10.004 22019719 PMC3289735

[B41] Kim J.-H. Auerbach J. M. Rodríguez-Gómez J. A. Velasco I. Gavin D. Lumelsky N. (2002). Dopamine neurons derived from embryonic stem cells function in an animal model of Parkinson’s disease. *Nature* 418 50–56. 10.1038/nature00900 12077607

[B42] Kirkeby A. Nolbrant S. Tiklova K. Heuer A. Kee N. Cardoso T. (2017). Predictive markers guide differentiation to improve graft outcome in clinical translation of hESC-based therapy for Parkinson’s disease. *Cell Stem Cell* 20 135–148. 10.1016/j.stem.2016.09.004 28094017 PMC5222722

[B43] Kordower J. H. Freeman T. B. Snow B. J. Vingerhoets F. J. G. Mufson E. J. Sanberg P. R. (1995). Neuropathological evidence of graft survival and striatal reinnervation after the transplantation of fetal mesencephalic tissue in a patient with Parkinson’s disease. *N. Engl. J. Med.* 332 1118–1124. 10.1056/NEJM199504273321702 7700284

[B44] Kordower J. H. Goetz C. G. Chu Y. Halliday G. M. Nicholson D. A. Musial T. F. (2017). Robust graft survival and normalized dopaminergic innervation do not obligate recovery in a Parkinson disease patient. *Ann. Neurol.* 81 46–57. 10.1002/ana.24820 27900791 PMC5890810

[B45] Kramer E. R. Liss B. (2015). GDNF–Ret signaling in midbrain dopaminergic neurons and its implication for Parkinson disease. *FEBS Lett.* 589 3760–3772. 10.1016/j.febslet.2015.11.006 26555190

[B46] Kriks S. Shim J.-W. Piao J. Ganat Y. M. Wakeman D. R. Xie Z. (2011). Dopamine neurons derived from human ES cells efficiently engraft in animal models of Parkinson’s disease. *Nature* 480 547–551. 10.1038/nature10648 22056989 PMC3245796

[B47] Lamas N. J. Johnson-Kerner B. Roybon L. Kim Y. A. Garcia-Diaz A. Wichterle H. (2014). Neurotrophic requirements of human motor neurons defined using amplified and purified stem cell-derived cultures. *PLoS One* 9:e110324. 10.1371/journal.pone.0110324 25337699 PMC4206291

[B48] Lee S. H. Lumelsky N. Studer L. Auerbach J. M. McKay R. D. (2000). Efficient generation of midbrain and hindbrain neurons from mouse embryonic stem cells. *Nat. Biotechnol.* 18 675–679. 10.1038/76536 10835609

[B49] Lin L. F. Doherty D. H. Lile J. D. Bektesh S. Collins F. (1993). GDNF: a glial cell line-derived neurotrophic factor for midbrain dopaminergic neurons. *Science* 260 1130–1132. 10.1126/science.8493557 8493557

[B50] Lindvall O. Widner H. Rehncrona S. Brundin P. Odin P. Gustavii B. (1992). Transplantation of fetal dopamine neurons in Parkinson’s disease: one-year clinical and neurophysiological observations in two patients with putaminal implants. *Ann. Neurol.* 31 155–165. 10.1002/ana.410310206 1575454

[B51] Lloyd K. G. Davidson L. Hornykiewicz O. (1975). The neurochemistry of Parkinson’s disease: effect of L-dopa therapy. *J. Pharmacol. Exp. Ther.* 195 453–464. 489

[B52] Marquardt L. M. Ee X. Iyer N. Hunter D. Mackinnon S. E. Wood M. D. (2015). Finely tuned temporal and spatial delivery of GDNF promotes enhanced nerve regeneration in a long nerve defect model. *Tissue Eng. Part A* 21 2852–2864. 10.1089/ten.tea.2015.0311 26466815 PMC4684669

[B53] Meka D. P. Müller-Rischart A. K. Nidadavolu P. Mohammadi B. Motori E. Ponna S. K. (2015). Parkin cooperates with GDNF/RET signaling to prevent dopaminergic neuron degeneration. *J. Clin. Invest.* 125 1873–1885. 10.1172/jci79300 25822020 PMC4611569

[B54] Meyer M. Matarredona E. R. Seiler R. W. Zimmer J. Widmer H. R. (2001). Additive effect of glial cell line-derived neurotrophic factor and neurotrophin-4/5 on rat fetal nigral explant cultures. *Neuroscience* 108 273–284. 10.1016/S0306-4522(01)00418-3 11734360

[B55] Mogi M. Togari A. Kondo T. Mizuno Y. Kogure O. Kuno S. (2001). Glial cell line-derived neurotrophic factor in the substantia nigra from control and parkinsonian brains. *Neurosci. Lett.* 300 179–181. 10.1016/s0304-3940(01)01577-4 11226640

[B56] Mogi M. Togari A. Kondo T. Mizuno Y. Komure O. Kuno S. (1999). Brain-derived growth factor and nerve growth factor concentrations are decreased in the substantia nigra in Parkinson’s disease. *Neurosci. Lett.* 270 45–48. 10.1016/s0304-3940(99)00463-2 10454142

[B57] Nasrolahi A. Mahmoudi J. Akbarzadeh A. Karimipour M. Sadigh-Eteghad S. Salehi R. (2018). Neurotrophic factors hold promise for the future of Parkinson’s disease treatment: is there a light at the end of the tunnel? *Rev. Neurosci.* 29 475–489. 10.1515/revneuro-2017-0040 29305570

[B58] Neuhoff H. Neu A. Liss B. Roeper J. (2002). I(h) channels contribute to the different functional properties of identified dopaminergic subpopulations in the midbrain. *J. Neurosci.* 22 1290–1302. 10.1523/JNEUROSCI.22-04-01290.2002 11850457 PMC6757558

[B59] Olanow C. W. Bartus R. T. Baumann T. L. Factor S. Boulis N. Stacy M. (2015). Gene delivery of neurturin to putamen and substantia nigra in Parkinson disease: a double-blind, randomized, controlled trial. *Ann. Neurol.* 78 248–257. 10.1002/ana.24436 26061140

[B60] Olanow C. W. Goetz C. G. Kordower J. H. Stoessl A. J. Sossi V. Brin M. F. (2003). A double-blind controlled trial of bilateral fetal nigral transplantation in Parkinson’s disease. *Ann Neurol.* 54 403–414. 10.1002/ana.10720 12953276

[B61] Pascual A. Hidalgo-Figueroa M. Piruat J. I. Pintado C. O. Gómez-Díaz R. López-Barneo J. (2008). Absolute requirement of GDNF for adult catecholaminergic neuron survival. *Nat. Neurosci.* 11 755–761. 10.1038/nn.2136 18536709

[B62] Perez-Bouza A. Di Santo S. Seiler S. Meyer M. Andereggen L. Huber A. (2017). Simultaneous transplantation of fetal ventral mesencephalic tissue and encapsulated genetically modified cells releasing GDNF in a hemi-parkinsonian rat model of Parkinson’s disease. *Cell Transplant.* 26 1572–1581. 10.1177/0963689717721202 29113462 PMC5680950

[B63] Piccini P. Brooks D. J. Björklund A. Gunn R. N. Grasby P. M. Rimoldi O. (1999). Dopamine release from nigral transplants visualized *in vivo* in a Parkinson’s patient. *Nat. Neurosci.* 2 1137–1140. 10.1038/16060 10570493

[B64] Poewe W. Seppi K. Tanner C. M. Halliday G. M. Brundin P. Volkmann J. (2017). Parkinson disease. *Nat. Rev. Dis. Prim.* 3:17013. 10.1038/nrdp.2017.13 28332488

[B65] Rodríguez-Gómez J. A. Lu J.-Q. Velasco I. Rivera S. Zoghbi S. S. Liow J.-S. (2007). Persistent dopamine functions of neurons derived from embryonic stem cells in a rodent model of Parkinson disease. *Stem Cells* 25 918–928. 10.1634/stemcells.2006-0386 17170065 PMC4151324

[B66] Roeper J. (2013). Dissecting the diversity of midbrain dopamine neurons. *Trends Neurosci.* 36 336–342. 10.1016/j.tins.2013.03.003 23582338

[B67] Rossi J. Tomac A. Saarma M. Airaksinen M. S. (2000). Distinct roles for GFRα1 and GFRα2 signalling in different cranial parasympathetic ganglia *in vivo*. *Eur. J. Neurosci.* 12 3944–3952. 10.1046/j.1460-9568.2000.00292.x 11069590

[B68] Santosa K. B. Jesuraj N. J. Viader A. MacEwan M. Newton P. Hunter D. A. (2013). Nerve allografts supplemented with schwann cells overexpressing glial-cell-line-derived neurotrophic factor. *Muscle Nerve* 47 213–223. 10.1002/mus.23490 23169341 PMC3556217

[B69] Sauer H. Rosenblad C. Björklund A. (1995). Glial cell line-derived neurotrophic factor but not transforming growth factor beta 3 prevents delayed degeneration of nigral dopaminergic neurons following striatal 6-hydroxydopamine lesion. *Proc. Natl. Acad. Sci. U. S. A.* 92 8935–8939. 10.1073/pnas.92.19.8935 7568047 PMC41082

[B70] Tenenbaum L. Humbert-Claude M. (2017). Glial cell line-derived neurotrophic factor gene delivery in parkinson’s disease: a delicate balance between neuroprotection, trophic effects, and unwanted compensatory mechanisms. *Front. Neuroanat.* 11:29. 10.3389/fnana.2017.00029 28442998 PMC5385337

[B71] Tripathy D. Haobam R. Nair R. Mohanakumar K. P. (2013). Engraftment of mouse embryonic stem cells differentiated by default leads to neuroprotection, behaviour revival and astrogliosis in parkinsonian rats. *PLoS One* 8:e72501. 10.1371/journal.pone.0072501 24069147 PMC3772067

[B72] Ungerstedt U. (1968). 6–Hydroxy–dopamine induced degeneration of central monoamine neurons. *Eur. J. Pharmacol.* 5 107–110. 10.1016/0014-2999(68)90164-7 5718510

[B73] Ungerstedt U. Arbuthnott G. W. (1970). Quantitative recording of rotational behavior in rats after 6-hydroxy-dopamine lesions of the nigrostriatal dopamine system. *Brain Res.* 24 485–493. 10.1016/0006-8993(70)90187-3 5494536

[B74] Wang J. Hu W.-W. Jiang Z. Feng M.-J. (2020). Advances in treatment of neurodegenerative diseases: perspectives for combination of stem cells with neurotrophic factors. *World J. Stem Cells* 12 323–338. 10.4252/wjsc.v12.i5.323 32547681 PMC7280867

[B75] Whone A. Boca M. Luz M. Woolley M. Mooney L. Dharia S. (2019a). Extended treatment with glial cell line-derived neurotrophic factor in Parkinson’s disease. *J. Parkinsons Dis.* 9 301–313. 10.3233/jpd-191576 30829619 PMC6597995

[B76] Whone A. Luz M. Boca M. Woolley M. Mooney L. Dharia S. (2019b). Randomized trial of intermittent intraputamenal glial cell line-derived neurotrophic factor in Parkinson’s disease. *Brain* 142 512–525. 10.1093/brain/awz023 30808022 PMC6391602

[B77] Widmer H. R. (2018). Combination of cell transplantation and glial cell line-derived neurotrophic factor–secreting encapsulated cells in Parkinson’s disease. *Brain Circ.* 4 114–117. 10.4103/bc.bc_19_18 30450417 PMC6187948

[B78] Wyse R. D. Dunbar G. L. Rossignol J. (2014). Use of genetically modified mesenchymal stem cells to treat neurodegenerative diseases. *Int. J. Mol. Sci.* 15 1719–1745. 10.3390/ijms15021719 24463293 PMC3958818

[B79] Xiao H. Hirata Y. Isobe K.-I. Kiuchi K. (2002). Glial cell line-derived neurotrophic factor up-regulates the expression of tyrosine hydroxylase gene in human neuroblastoma cell lines. *J. Neurochem.* 82 801–808. 10.1046/j.1471-4159.2002.00993.x 12358785

[B80] Yang F. Feng L. Zheng F. Johnson S. W. Du J. Shen L. (2001). GDNF acutely modulates excitability and A-type K(+) channels in midbrain dopaminergic neurons. *Nat. Neurosci.* 4 1071–1078. 10.1038/nn734 11593232

[B81] Yang X. Mertens B. Lehtonen E. Vercammen L. Bockstael O. Chtarto A. (2009). Reversible neurochemical changes mediated by delayed intrastriatal glial cell line-derived neurotrophic factor gene delivery in a partial Parkinson’s disease rat model. *J. Gene Med.* 11 899–912. 10.1002/jgm.1377 19639608

[B82] Yurek D. M. (1998). Glial cell line-derived neurotrophic factor improves survival of dopaminergic neurons in transplants of fetal ventral mesencephalic tissue. *Exp. Neurol.* 153 195–202. 10.1006/exnr.1998.6884 9784279

[B83] Zurn A. D. Widmer H. R. Aebischer P. (2001). Sustained delivery of GDNF: towards a treatment for Parkinson’s disease. *Brain Res. Rev.* 36 222–229. 10.1016/S0165-0173(01)00098-4 11690619

